# Coxsackievirus B infections are common in Cystic Fibrosis and experimental evidence supports protection by vaccination

**DOI:** 10.1016/j.isci.2022.105070

**Published:** 2022-09-05

**Authors:** Virginia M. Stone, Renata Utorova, Marta Butrym, Amir-Babak Sioofy-Khojine, Minna M. Hankaniemi, Emma E. Ringqvist, Marfa Blanter, Anirudra Parajuli, Terezia Pincikova, Björn Fischler, Ferenc Karpati, Vesa P. Hytönen, Heikki Hyöty, Lena Hjelte, Malin Flodström-Tullberg

**Affiliations:** 1 Center for Infectious Medicine, Department of Medicine Huddinge, Karolinska Institutet and Karolinska University Hospital Huddinge, 141 52 Stockholm, Sweden; 2 Faculty of Medicine and Health Technology, Tampere University, 33014 Tampere, Finland; 3 Stockholm CF Center, Karolinska University Hospital Huddinge, 141 86 Stockholm, Sweden; 4 Division of Pediatrics, Department of Clinical Science, Intervention and Technology, Karolinska Institutet and Department of Pediatrics, Karolinska University Hospital, 141 86 Stockholm, Sweden; 5 Fimlab Laboratories, 33520 Tampere, Finland

**Keywords:** Virology

## Abstract

Viral respiratory tract infections exacerbate airway disease and facilitate life-threatening bacterial colonization in cystic fibrosis (CF). Annual influenza vaccination is recommended and vaccines against other common respiratory viruses may further reduce pulmonary morbidity risk. Enteroviruses have been found in nasopharyngeal samples from CF patients experiencing pulmonary exacerbations. Using serology tests, we found that infections by a group of enteroviruses, Coxsackievirus Bs (CVBs), are prevalent in CF. We next showed that a CVB vaccine, currently undergoing clinical development, prevents infection and CVB-instigated lung damage in a murine model of CF. Finally, we demonstrate that individuals with CF have normal vaccine responses to a similar, commonly used enterovirus vaccine (inactivated poliovirus vaccine). Our study demonstrates that CVB infections are common in CF and provides experimental evidence indicating that CVB vaccines could be efficacious in the CF population. The role of CVB infections in contributing to pulmonary exacerbations in CF should be further studied.

## Highlights

• CVB infections are common in CF
• A CVB vaccine prevents infection and tissue damage in a model of CF
• Most people with CF have robust antibody responses to a similar enterovirus vaccine

## Graphical abstract

**Figure:**
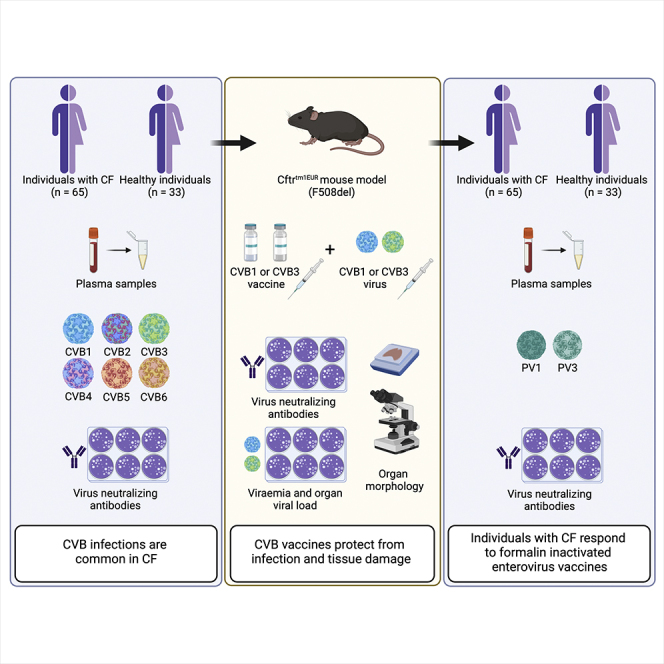

## Introduction

Cystic fibrosis (CF) is caused by mutations in the cystic fibrosis transmembrane regulator (*CFTR*) gene that encodes a chloride channel. Of 2,000 + CFTR mutations identified, the most common is F508del (the deletion of phenylalanine at position 508). CFTR is expressed in most organs with a secretory epithelium including the sinuses, respiratory system, pancreas, and reproductive organs, as well as immune cells (Elborn, 2016; Marson et al., 2016).

Acute and chronic airway infections are the major cause of mortality and morbidity in CF. Most patients are intermittently or chronically colonized by pathogenic airway bacteria that cause chronic airway inflammation and successive decline in lung function (Elborn, 2016). Why individuals with CF suffer from these infections remains unknown. Numerous observations suggest respiratory virus infections are a major cause of pulmonary exacerbations in chronic lung disease and predispose the CF airway to bacterial colonization (Asner et al., 2012; de Almeida et al., 2010; Goffard et al., 2014; Johansen and Hoiby, 1992; Petersen et al., 1981; Singanayagam et al., 2012; Wat et al., 2008). Such exacerbations lead to progressive worsening of CF lung function and culminate in terminal lung disease, the leading cause of death in CF.

Viral pathogens commonly associated with pulmonary exacerbations in CF include enteroviruses, influenza, and respiratory syncytial virus (Asner et al., 2012; de Almeida et al., 2010; Eymery et al., 2019). The frequency and seasonality of respiratory virus infections in CF populations do not seemingly differ from healthy individuals; however, the symptom duration is longer in children with CF (Dijkema et al., 2016; van Ewijk et al., 2008). Individuals with virus-associated pulmonary exacerbations do, however, require prolonged antibiotic treatment and they respond inadequately to standard therapeutic interventions (Etherington et al., 2014). Therefore, preventing common viral infections is likely to be of significant benefit to lung function and survival in CF.

Enteroviruses are common single-stranded RNA viruses. They primarily infect mucosal tissue in the respiratory and intestinal tracts and include rhinoviruses, echoviruses, polioviruses, and Coxsackie A and B viruses. These have all (with the exception of polioviruses) been detected in nasopharyngeal samples from patients with CF, especially those experiencing pulmonary exacerbations (de Almeida et al., 2010; Dijkema et al., 2016; Dowaikh et al., 2017; Goffard et al., 2014; Hamed et al., 2022; Ong et al., 1989; van Ewijk et al., 2008). Enterovirus vaccines could therefore help to reduce respiratory virus infections in CF.

At present, there are only a few enterovirus vaccines available (poliovirus vaccines and EV71 vaccines, the latter is only approved for clinical use in Asia) and none against viruses linked to pulmonary decline in CF. Recent vaccine efforts (Hankaniemi et al., 2017; Hyoty et al., 2018; Larsson et al., 2015; Stone et al., 2018, 2020) have however resulted in the commencement of phase I human trials (NCT04690426) with a vaccine against a group of common enteroviruses, the Coxsackie B viruses (CVBs). Common symptoms caused by the CVBs (serotypes 1–6) are fever, headaches, a sore throat, fatigue, and chest and muscle pain. More severe infections can lead to serious diseases like pleurodynia (Bornholm disease), hepatitis, and myocarditis (Marjomaki and Flodstrom-Tullberg, 2022), and CVBs are a suspected causal agent in type 1 diabetes (Richardson and Morgan, 2018). Infections are common and mainly occur in childhood, but can also happen in adulthood. CVB infections have been reported in CF (Dowaikh et al., 2017; Ong et al., 1989). Moreover, we have found that F508del mice (a murine model of CF) infected with CVB serotype B3 (CVB3) have increased morbidity and mortality compared with wild-type animals. This was accompanied by increased viral loads in the lungs of the F508del mice (Svedin et al., 2017). Whether CVB infections are common in CF has not been studied. However, if prevalent in this population, the CVB vaccine could be used prophylactically to prevent infections, thereby contributing to a lowering in the frequency of virus infections that cause upper respiratory tract symptoms.

Alterations in the innate and adaptive arms of the immune system have been described in CF, which may affect vaccination responses (Giacalone et al., 2020; Lara-Reyna et al., 2020). Divergent humoral responses have been reported including occasional hypogammaglobulinemia (Matthews et al., 1980), impaired antibody responses to polysaccharide antigens (Browning et al., 2014; Moss et al., 1987), and IgG subclass deficiency (Browning et al., 2014; Garside et al., 2005, 2007). We have also reported that mice harboring the F508del mutation have an impaired immune response to CVB3 infection, with defective viral clearance linked to a delay in virus-neutralizing antibody (nAB) production (Svedin et al., 2017). Despite these observations, few studies have been conducted assessing vaccine responses and virus nAB durability in CF (Browning et al., 2014; Hostetler et al., 2021; Launay et al., 2014; Lucidi et al., 1996).

In this study, we examined the prevalence of CVB infections in CF and compared this with a cohort of healthy controls. Our findings suggest that CVB infections are common in CF. We then performed proof-of-concept studies in a pre-clinical CF model examining protection against CVB infection and CVB-induced tissue pathology after vaccination with a CVB vaccine. Finally, we assessed immunity against poliovirus in CF and healthy control cohorts to gain insight into nAB responses to a similar, commonly used enterovirus vaccine.

## Results

### CVB infections are frequent in CF

To assess whether CVB infections are common in CF and compare their frequency with a healthy population we used serum samples collected from individuals that attended the Stockholm CF Center, Stockholm, Sweden (Table 1). The control group consisted of healthy volunteers who participated in separate vaccine studies at the Karolinska Institute, Stockholm, Sweden (Table 1). Individuals with CF (*n* = 65) were on average younger than the healthy controls (*n* = 33) (*p* < 0.05), but the female-to-male ratio was not significantly different. All individuals with CF had a classic CF phenotype (defined as one or more clinical phenotype characteristic(s) and a sweat chloride concentration of >60 mmol/L) and 60% were homozygous for the F508del mutation.

**Table 1:** Characteristics of the CF patient and control cohorts

Characteristic	CF patients (*n* = 65)	Controls (*n* = 33)
Age mean ± SD (min – max)	22.5 ± 12 (7–44)	27.0 ± 8 (18–55)
Age under 18 (%)	30 (40)	0 (0)
Female/male (%/%)	30/35 (46/54)	21/12 (64/36)
Genotype F508del/F508del (%)	39 (60)	n/a
CF phenotype, classic/non-classic (%/%)	65/0 (100/0)	n/a
SD = standard deviation, n/a = not applicable		

**Figure 1:**
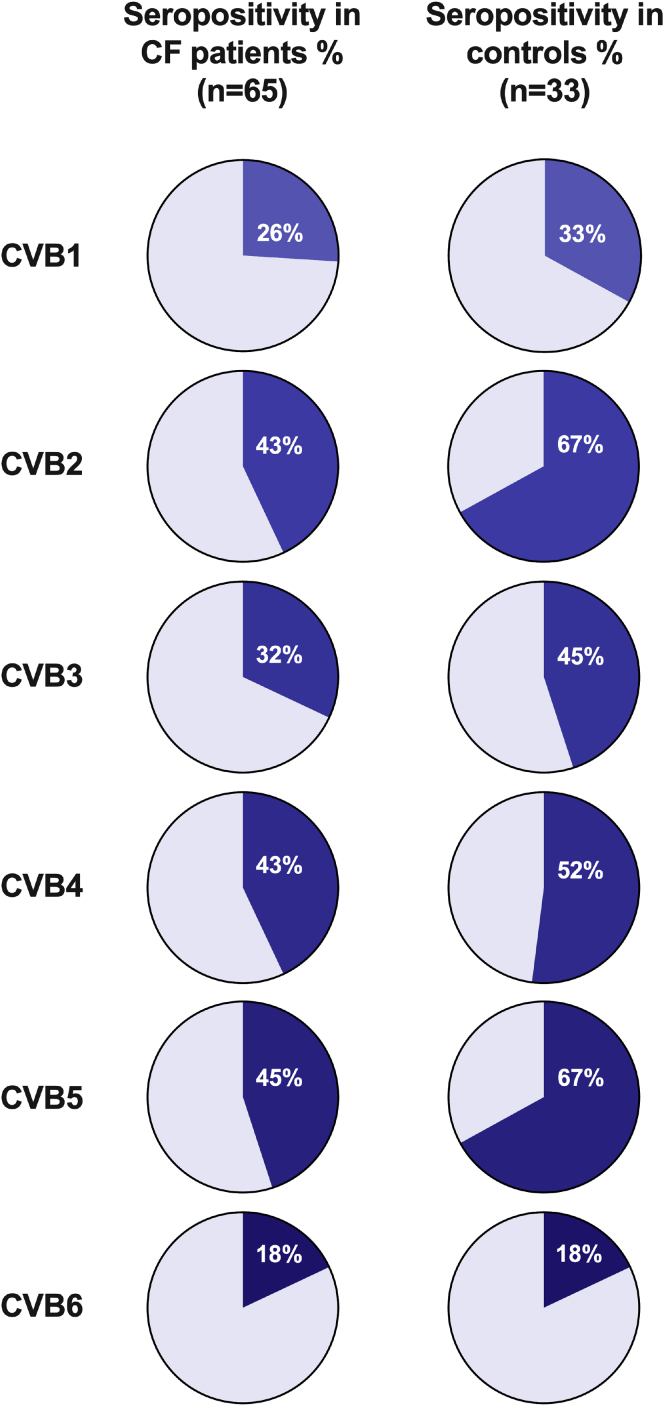
CVB1-6 seropositivity in CF and healthy control cohorts Serum was extracted from blood-samples collected at yearly check-up appointments in the CF cohort (*n* = 65) or from samples taken from healthy individuals (*n* = 33) included in other studies at the Karolinska Institute. CVB1-6 seropositivity was examined by measuring the presence of neutralizing antibodies against the CVB1-6 viruses, using a standard plaque reduction assay. The cut-off titer for seropositivity was set to ≥1:16 for all six serotypes. The pie charts show the fraction of individuals that were positive for each CVB serotype in the two groups (blue color) and the white numbers show the exact percentage of positive cases in each group. See also Figure S1.

Analysis of serum samples for CVB1-6 nABs revealed the serotypes participants were previously infected by. Most individuals with CF (89%) and all healthy controls (100%) were seropositive for at least one CVB serotype. On average, individuals with CF had encountered 2.1 ± 1.5 (2) (mean ± SD (median)) CVB serotypes compared with 2.8 ± 1.4 (3) for healthy individuals. CVB5 was the most common serotype in individuals with CF, whilst CVB2 and CVB5 had the highest frequencies in the healthy controls (Figures 1 and S1). As the individuals with CF were on average younger than the healthy control group (Table 1) and the latter consisted solely of individuals aged 18 or above, we next grouped the individuals with CF into those aged below 18 years (*n* = 30, 12.2 ± 3.6 years) and those 18 years old or above (*n* = 35; 31.4 ± 8.4 years). The average number of CVB serotypes that these individuals had been exposed to were (mean ± SD) for age group <18 years, 1.8 ± 1.4, and for age group ≥18 years, 2.3 ± 1.5. These results suggest that CVB infections are common in CF with a similar prevalence to healthy subjects.

### CVB vaccines induce virus nABs in an experimental mouse model of CF

**Figure 2:**
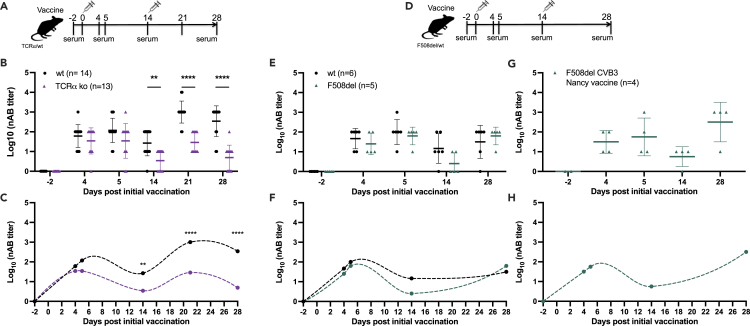
Immune response to a CVB3 vaccine is in part T-cell-dependent but is mainly intact in F508del mice (A–C) Female TCRα ko (purple triangles; *n* = 13) and wt (black circles; *n* = 14) mice were vaccinated on two occasions (days 0 and 14) with a CVB3-field isolate vaccine (1.8 μg per dose, interscapular injection). (A) Experimental setup. (B) CVB3 neutralizing antibody (nAB) titers measured in the serum on days −2, 4, 5, 14, 21, and 28 after the initial vaccination by standard plaque neutralization assay. Individual mice are represented by single symbols. The horizontal line represents the mean ± SD ∗∗*p* < 0.01, ∗∗∗∗*p* < 0.0001 by two-way ANOVA with Sidak’s multiple comparison test. (C) nAB titer fluctuations after vaccination in the wt and TCRα KO mice visualized by an Akima spline. (D–F) Female F508del (green triangles; *n* = 5) and wt (black circles; *n* = 6) mice were vaccinated with a CVB3-field isolate vaccine (also used in a-c; 1.8 μg per dose, interscapular injection) on two occasions (days 0 and 14) as illustrated in (D). ICVB3 nAB titers were measured in the serum on days −2, 4, 5, 14, and 28 after the initial vaccination by standard plaque reduction assay. Individual mice are represented by single symbols. The horizontal line represents the mean ± SD. No statistical differences in nAB titers were found between the wt and F508del mice, two-way ANOVA with Sidak’s multiple comparison test. (F) Virus nAB titer fluctuations after vaccination with CVB3-field isolate vaccine in the wt and F508del mice visualized by an Akima spline. (G) Female and male F508del mice were vaccinated with CVB3-Nancy vaccine (*n* = 4; green triangles; 1.8 μg per dose, interscapular injection) twice on days 0 and 14 as illustrated in (D). CVB3 nAB titers measured in the serum on days −2, 4, 5, 14, and 28 after the initial vaccination by standard plaque neutralization assay. Individual mice are represented by single symbols. The horizontal line represents the mean ± SD. (H) nAB titer fluctuations after vaccination with CVB3-Nancy vaccine in F508del mice visualized by an Akima spline. See also Figures S2 and S3.

Vaccination against CVB viruses could constitute an attractive complementary therapy to reduce the respiratory virus infection burden in CF, therefore we next explored the feasibility of this concept. Various CF animal models exist (Semaniakou et al., 2018), including the Cftr^tm1EUR^ mouse model that is on a C57Bl/6J background and harbors the most common mutation, F508del (hereon referred to as F508del mice) (van Doorninck et al., 1995). Previously we performed side-by-side comparisons of infected wild-type and F508del mice and reported that F508del mice have a delayed nAB response to live CVB3 virus, which was linked in part to a defective antibody response to T cell-dependent antigens (Svedin et al., 2017). Therefore, we investigated whether the nAB response to CVB vaccination is T cell-dependent using mice lacking TCRαβ T-cells (TCRα knock-out, ko, representative plot Figure S2) and their wild-type (wt) counterparts. Mice were vaccinated twice (days 0 and 14) with a monovalent CVB3 vaccine (field isolate strain, (Laitinen et al., 2014); Figure 2A). The CVB3 vaccine was well tolerated (Figure S3A, data not shown) and from day 14, nAB titers were significantly lower in TCRα-ko mice compared with the wt animals (*p* < 0.001; Figures 2B and 2C) indicating that nAB responses to a CVB vaccine are in part T cell-dependent.

We next tested whether F508del mice produce virus nABs in response to CVB vaccination. A two-dose vaccination schedule (days 0 and 14; Figure 2D) was employed and F508del and wild-type littermate control mice (wt; C57Bl/6J) were monitored until day 28 after the initial vaccination. Most mice tolerated the CVB3 vaccine well as indicated by good health status scores throughout the study (data not shown). However, after the first vaccination, two F508del mice were removed after losing more weight than was allowed by our ethical permit (>10%; data not shown; these animals were excluded from the analysis in Figure 2). We have not observed such weight loss in our previous studies where we vaccinated mice with varying genetic backgrounds and non-human primates (Hankaniemi et al., 2017; Larsson et al., 2015; Stone et al., 2018, 2020). Occasional deaths have been reported by others that use F508del mice and the expected survival to maturity with this model is 90%. Similar or lower survival rates are seen in other murine models of CF (Semaniakou et al., 2018). Based on this we do not believe that the weight loss of the two described animals was a vaccine-related event.

**Figure 3:**
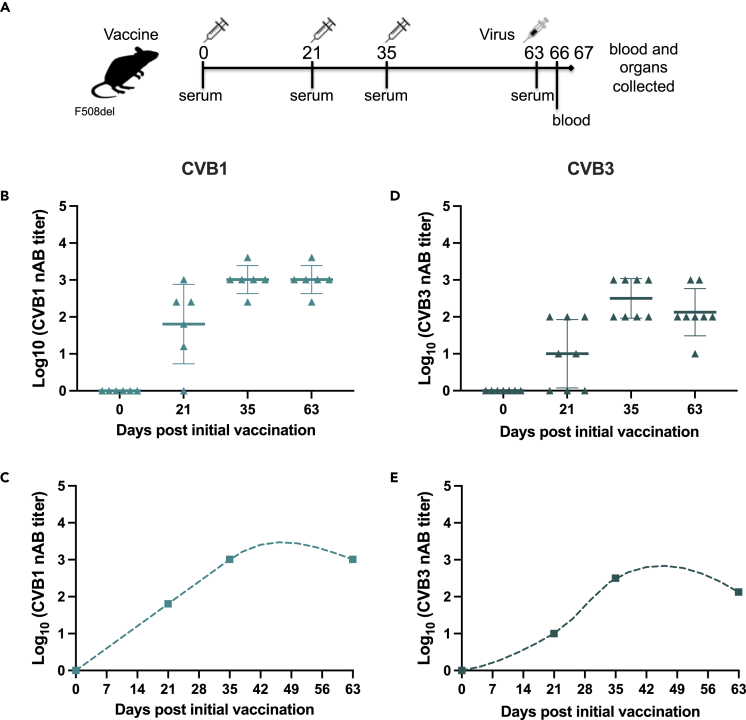
F508del mice raise virus neutralizing antibodies when vaccinated with monovalent CVB1 and CVB3 vaccines Female and male F508del mice were left untreated (*n* = 1, CVB3 study), mock-vaccinated with buffer (*n* = 3 for CVB3, *n* = 6 for CVB1), or vaccinated three times (on days 0, 21, and 35) with either CVB1 (CDC7; *n* = 6) vaccine (B and C), or CVB3 (Nancy; *n* = 8) vaccine (D and E), as illustrated by the vaccination schedule illustrated in (A). Each vaccine dose contained 1.8 μg of protein and was given by interscapular injection. The animals were infected with either CVB1 or CVB3 virus on day 63 after the initial vaccine dose and monitored until day 67. (B and D) Virus neutralizing antibody (nAB) titers in the serum of mice vaccinated with CVB1 vaccine (D; light teal triangle) or CVB3 vaccine (e; dark green triangle) on days 0, 21, 35, and 63 after the prime vaccination as measured by standard plaque neutralization assay. Individual animals are represented by individual symbols. The horizontal line represents the mean ± SD. (C and E) Fluctuations in virus neutralizing antibodies in CVB1- (C) and CVB3- (E) vaccinated mice as visualized by Akima Spline. See also Figure S4.

No further adverse consequences on weight were recorded for mice in the study (Figure S3B). Wt and F508del mice raised CVB3 nABs after the first vaccination with no statistical differences in nAB titers on days 4 and 5 (Figures 2E and 2F). By day 14, F508del mice had lower CVB3 nAB titers compared with wt mice although the difference was not statistically significant (Figures 2E and 2F). After the day 14 boost vaccination, CVB3 nAB titers were equivalent between wt and F508del mice (Figures 2E and 2F). To confirm these studies, F508del mice were vaccinated with another CVB3 vaccine that was produced in an identical manner to the initial vaccine but utilized a different CVB3 virus strain (the Nancy strain; Figures 3G and 3H). This vaccine was also well tolerated and induced a similar nAB response to the first vaccine (Figures 2G, 2H, and S3C). These data show that F508del mice are as capable as wt mice of raising virus nABs after a booster vaccination.

### Systemic CVB infections and viral spread to organs, including the lung, are prevented in F508del immunized with CVB vaccines

**Figure 4:**
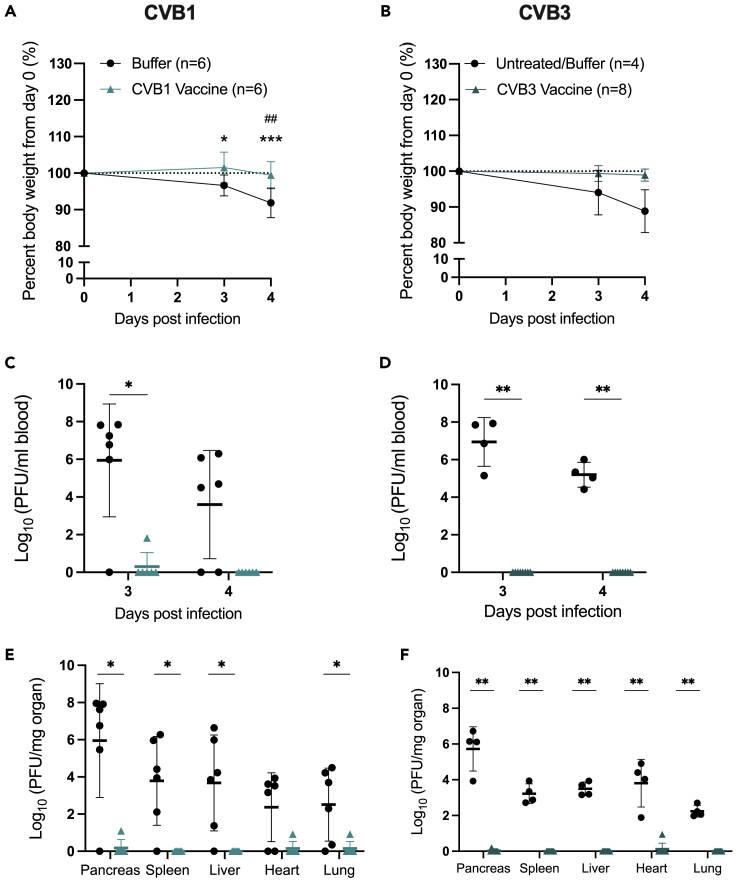
CVB vaccines prevent acute CVB infections in F508del mice Female and male F508del mice were left untreated (*n* = 1, CVB3 study; black circles), mock-vaccinated with vaccine buffer (*n* = 6, CVB1 study; *n* = 3 CVB3 study; black circles), or vaccinated with CVB1 vaccine (A, C, and E; *n* = 6; light teal triangles) or CVB3 vaccine (B, D, and F; *n* = 8; dark teal triangles) as depicted in the schematic in Figure 3A. On day 63 after the prime vaccination, the animals were infected with CVB1 (A, C, and E) or CVB3 (B, D, and F) and blood was collected on days 3 and 4 post-infection (p.i.). The experiment was terminated on day 4 p.i. and organs were collected. (A and B) Mean percentage body weight from the day of infection (day 0) in buffer-treated and CVB-vaccinated mice. Shown are the mean values ±SD ∗*p* < 0.05 and ∗∗∗*p* < 0.001 when comparing buffer versus vaccine at each time point by two-way ANOVA with Sidak’s multiple comparison test. ##*p* < 0.01 when comparing the time point with day 0 in each group by two-way ANOVA with Sidak’s multiple comparison test. (C and D) Replicating virus in the blood of untreated/buffer-treated, CVB1-vaccinated (C), or CVB3-vaccinated (D) mice as measured by standard plaque assay on days 3 and 4 p.i.. Each animal is represented by an individual symbol and the horizontal line represents the mean ± SD ∗*p* < 0.05 and ∗∗*p* < 0.01 as compared by the Mann–Whitney U test. (E and F) Titers of replicating virus particles in the organs of untreated/buffer-treated, CVB1-vaccinated (E), or CVB3-vaccinated (F) mice on day 4 p.i. measured by a standard plaque assay. Each animal is represented by an individual symbol and the horizontal line represents the mean ± SD ∗*p* < 0.05 and ∗∗*p* < 0.01 as compared by the Mann–Whitney U test.

We have previously shown that CVB vaccines protect C57Bl/6J mice and other mouse strains from CVB infections (Stone et al., 2018, 2020, 2021). Next, we examined whether CVB vaccines also protect F508del mice against CVB infections. A CVB1 vaccine was also included and the vaccination strategy optimized to a three-dose vaccination strategy to increase the duration of the virus nAB response (Figure 3A; days 0, 21 and 35) (Hankaniemi et al., 2017; Larsson et al., 2015; Stone et al., 2018, 2020). F508del mice gained weight normally after vaccination (Figures S4A and S4B) and had robust CVB1 or CVB3 nAB responses nine weeks after the first vaccination when the mice were infected (Figures 3B–3E). Unvaccinated F508del mice infected with CVB1 and CVB3 lost weight by day 4 post-infection (p.i.; Figures 4A and 4B). Contrastingly, vaccinated mice were protected from virus-induced weight loss (Figures 4A and 4B). Infection also caused viraemia (replicating virus in the blood) on days 3 and 4 p.i. in 83% (5/6) and 100% (4/4) of the control (untreated or mock-vaccinated) F508del mice infected with CVB1 or CVB3, respectively (Figures 4C and 4D). Both vaccines completely prevented systemic infections at the same time points p.i. in all CVB1 (6/6) and CVB3- (8/8) vaccinated animals infected with their respective CVBs (Figure 4D).

Viral dissemination to peripheral organs was also assessed on day 4 p.i.. Most unvaccinated F508del mice infected with CVB1 (5/6; 83%; Figure 4E) or CVB3 (4/4; 100%; Figure 4F) had replicating virus in peripheral organs (virus was detected in the heart in 4/6 of CVB1-infected mice). Consistent with the viraemia data, all vaccinated mice challenged with CVBs were protected from viral dissemination to peripheral organs (Figures 4E and 4F).

### CVB vaccines prevent CVB-induced damage to lungs and other organs in F508del mice

**Figure 5:**
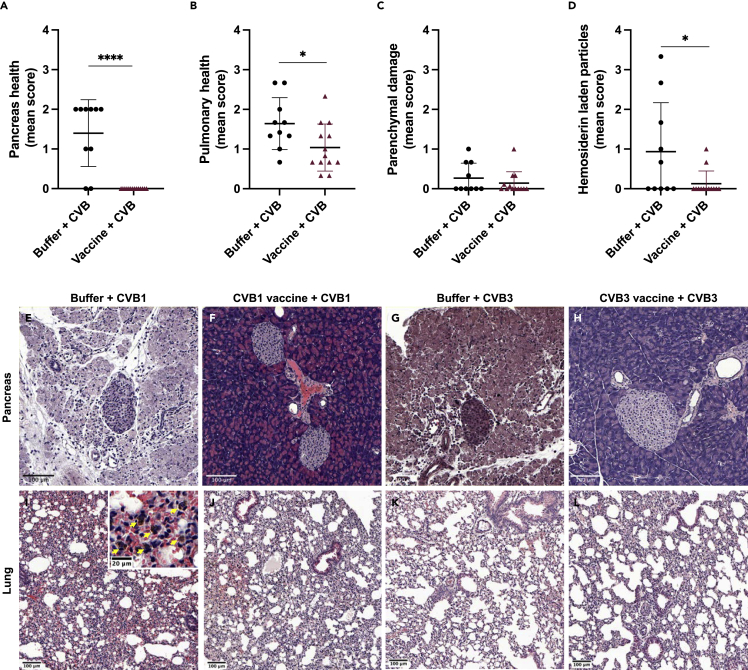
Vaccination of F508del mice with CVB vaccines prevent virus-mediated organ damage F508del mice were buffer-treated (*n* = 10) or vaccinated with CVB1 or CVB3 vaccines (*n* = 13) and infected with CVB1 or CVB3 virus, as shown in Figure 3A. Organs were collected on day 4 post-infection for histological analysis of organ integrity by haematoxylin and eosin (H&E) staining of formalin fixed paraffin embedded sections. (A) Pancreas scores of buffer + CVB or vaccine + CVB groups. Pancreas were scored according to exocrine damage and the presence of infiltrating immune cells. (B) Parenchymal health, (C) parenchymal damage and (D) hemosiderin laden particle staining in the lungs of buffer-treated + CVB-infected or vaccinated + CVB-infected mice. For (A)–(D), a score of 0 indicates healthy tissue and a score of 4 indicates highly damaged tissue. Each mouse is represented by an individual symbol and shown are the mean values ±SD ∗*p* < 0.05, ∗∗∗∗*p* < 0.001, as compared by the unpaired *t*-test. (E−L) Representative images of H&E-stained pancreas (E−H) and lung (I–L) from buffer + CVB1 (E and I), CVB1 vaccine + CVB1 (F and J), buffer + CVB3 (G and K), and CVB3 vaccine + CVB (H and L)-treated F508del mice on day 4 post-infection. In (I), the insert is a 5× magnification of the original image and the yellow arrows show hemosiderin laden particles. See also Figures S5 and S6.

Organs were histologically assessed for signs of virus-mediated damage. The pancreas is one of the first organs to incur CVB-mediated damage (e.g. Flodstrom et al., 2001). Exocrine tissue damage and pancreatitis (depicted as immune cell infiltration) were present in 83 and 75% of the CVB1- and CVB3-infected control mice, respectively. Contrastingly, all pancreas collected from vaccinated-infected mice were healthy in appearance (Figure 5A, representative images Figures 5E–5H, serotype breakdown Figures S5A and S5E). In all mice, the pancreatic islets of Langerhans remained intact (Figures 5E–5H), as previously described (e.g. Flodstrom et al., 2001).

Overall health scores of lung tissue were significantly worse in unvaccinated infected mice compared with vaccinated and infected animals suggesting CVB-induced tissue damage had occurred (*p* < 0.03; Figures 5B and S5B–S5F). A trend toward more parenchymal damage was seen in the lungs of control-infected mice compared with the vaccinated equivalents, although it was not statistically significant (Figures 5C, S5C, and S5G). Hemosiderin deposits, a sign of previous or ongoing wound healing (bleeding), were detected in the lungs of several mice (Figures 5D, 5I, and S5D). Deposits were found in the extracellular space and within macrophage-like cells in at least half of the unvaccinated animals but were rarely seen in vaccinated mice, with the former having a significantly higher mean hemosiderin deposit score after CVB infection (*p* < 0.04; Figure 5D). Most hemosiderin deposits were found in CVB1-infected animals rather than those challenged with CVB3 (Figures S5D and S5H).

Liver morphology also differed between the vaccinated and control groups after infection. Ballooning degeneration (Flodstrom et al., 2001) was seen in 50% of the control CVB3-infected animals (Figure S6C) but was absent in vaccinated mice (Figure S6D; data not shown). Furthermore, mild steatosis was detected in 83 and 75% of control mice infected with CVB1 and CVB3 respectively but was absent in the majority of vaccinated-infected mice (1/8 CVB3 vaccine + CVB3-challenge and 0/6 CVB1 vaccine + CVB1-challenge; representative image Figure S6A). Histological assessment of spleens did not reveal any noticeable morphological differences between the groups (data not shown).

Together, these results demonstrate that CVB vaccines protect F508del mice from acute CVB infections, viral dissemination to organs, and virus-mediated tissue damage, including in the lung.

### Most individuals with CF respond to poliovirus vaccination

CF is associated with altered immune functions (Giacalone et al., 2020; Lara-Reyna et al., 2020) and reports exist describing weakened vaccine responses in individuals with CF (Browning et al., 2014; Hostetler et al., 2021; Launay et al., 2014; Lucidi et al., 1996). A weak response to the CVB vaccine could limit the efficacy of future vaccine efforts in this group. One of the few enterovirus vaccines available, the inactivated poliovirus vaccine (IPV), is commonly used world-wide including in the Swedish childhood vaccination program. As poliovirus infections are most common in childhood, the vaccine is given early in life (one vaccine dose at each of the following ages: 3, 5, and 12 months), and after immunization, most children develop life-long protective virus nABs. IPV is based on inactivated whole virus and is produced in the same way as the newly developed CVB vaccines (Hankaniemi et al., 2017; Stone et al., 2018, 2020). As such, we hypothesized that the measurement of nAB titers to poliovirus 1 and 3 in our Swedish CF patient cohort and healthy controls would provide insight into how well individuals with CF respond to similar enterovirus vaccines.

**Figure 6:**
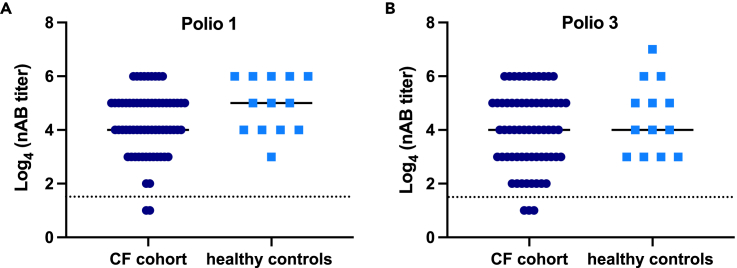
Individuals with CF mount a similar level of immunity to poliovirus vaccine as healthy controls Serum was collected from blood taken from CF patients at their annual check-up meeting (*n* = 65) or from healthy controls included in other studies at the Karolinska Institute (*n* = 33). Neutralizing antibodies (nAB) specific for polio 1 (A) and polio 3 (B) were measured by standard plaque reduction assay. The data are expressed as a log_4_ of 4-fold dilutions (1:4 to 1:16,384 dilutions). The solid lines represent the median values and the dotted lines represent the cut-off for protective antibody titers (≥1:8 for polio 1 and 3). No significant differences were observed between the two groups using an unpaired *t*-test.

Polio nAB titers were measured in the same cohorts used to assess CVB infection frequency. Titers of polio 1 and 3 nABs that correlated with protective immunity (seropositivity at a ≥1:8 dilution) were detected in most individuals with CF and healthy controls ((O’Ryan et al., 2015); Figures 6A and 6B). Individuals with CF tended to have lower nAB titers against both poliovirus serotypes but this was not statistically significant (Figures 6A and 6B). A small fraction of individuals with CF had titers below 1:8 (poliovirus 1: 2/65; poliovirus 3: 3/65). These data show that most individuals with CF respond well to a formalin-inactivated enterovirus vaccine.

## Discussion

Numerous studies have demonstrated that respiratory viruses, including enteroviruses, influence pulmonary exacerbations in CF (Asner et al., 2012; de Almeida et al., 2010; Goffard et al., 2014; Johansen and Hoiby, 1992; Petersen et al., 1981; Singanayagam et al., 2012; Wat et al., 2008). Enteroviruses have been found in pharyngeal samples of individuals with CF experiencing pulmonary exacerbations (de Almeida et al., 2010; Dijkema et al., 2016; Dowaikh et al., 2017; Goffard et al., 2014; Hamed et al., 2022; Ong et al., 1989; van Ewijk et al., 2008). Moreover, a recent cross-sectional observational study showed a significant relationship between sputum-positive bacterial culture and enterovirus positivity. A positive test for enterovirus or influenza virus but not rhino-, adeno-, Boca, or human metapneumovirus, was associated with a high risk of hospital admission and need for oxygen therapy (Hamed et al., 2022). These observations suggest that vaccination against enteroviruses would be a viable strategy to lower the burden of respiratory infections in CF.

The present study shows that infections by a group of enteroviruses, the CVBs, are common in CF. Little was known about CVB infection frequencies in individuals with CF and therefore serological assays were used to examine this using serum samples from individuals attending the Stockholm CF Center and healthy controls from the Stockholm region. The analyses revealed that CVB infections did not occur more frequently in CF patients compared with healthy individuals but rather we noted a slightly lower CVB seroprevalence in those with CF. This could be because those with CF often employ extra precautions to avoid respiratory infections. Alternatively, individuals with CF may have a weaker nAB response to CVB infection, causing some infections to be undetectable in this cohort. Nevertheless, in our study individuals had on average encountered at least two of the six CVB serotypes, suggesting that CVB infections are common in CF.

A CVB vaccine is currently in phase I clinical trials (2021). A superior method to determine vaccine efficacy and provide evidence of vaccine functionality involves examining protection against infection *in vivo.* Studies were therefore undertaken to assess the efficacy of CVB vaccines in a pre-clinical model of CF. CVB vaccination of F508del mice protected against virus-induced weight loss, viremia, and virus dissemination to peripheral organs. Moreover, histological tissue analyses revealed vaccine-mediated protection against CVB-induced pancreas and liver tissue damage. Most importantly, analysis of lung sections showed that vaccinated F508del mice had less damage compared with control F508del animals, particularly when assessing general health and assessing hemosiderin deposits that were more prevalent in control animals. CVB infections are in general associated with mild upper respiratory tract infections and only few studies have documented pulmonary involvement in humans and animal models following infection (e.g. Jahn et al., 1964; Wang et al., 2014). The mechanisms behind the pathophysiology seen in the lungs of the CVB1-infected F508del mice could be investigated in future studies with an aim to understand how mutations in *CFTR* heighten the risk of pulmonary disease. Collectively, our current data showed that vaccination protected F508del mice from virus-induced lung damage.

Alterations in immune system functions (reviewed in Giacalone et al., 2020; Lara-Reyna et al., 2020) and impaired vaccine responses (Browning et al., 2014; Hostetler et al., 2021; Launay et al., 2014; Lucidi et al., 1996) have been described in CF. Measuring nAB titers against polioviruses provides a reasonable surrogate to determine whether individuals with CF have an impaired immune response to enterovirus vaccines. In our study most individuals with CF had a strong, persistent nAB response to poliovirus vaccination (protective titers ≥1:8), however, a few individuals had low nAB titers (<1:8) to poliovirus 1 (2/65) and poliovirus 3 (3/65). This is consistent with the one existing study that examined immunity to the oral poliovirus vaccine in CF (Lucidi et al., 1996). Notably, it was observed that children >8 years old had weaker nAB responses to poliovirus 2, although titers always reached protective levels (Lucidi et al., 1996). Furthermore, another recent study described low or undetectable immunity to measles, mumps, and varicella zoster in a subgroup of individuals with CF (Hostetler et al., 2021). As CVB infections are common in childhood, immunizations with CVB vaccines would likely follow the same vaccination schedule as IPV (i.e. given in the first year of life). Our study shows that F508del mice generate similar CVB nAB titers to wt mice after a booster CVB vaccine dose, despite an initial weaker response, and that most individuals with CF responded adequately to IPV in early life. Taken together, one can hypothesize that the majority of individuals with CF should respond well to a CVB vaccine but tests confirming immunization-induced immunity should be routinely performed in this group.

Based on strong pre-clinical data including robust nAB responses in non-human primates (Stone et al., 2020), the development of a polyvalent formalin-inactivated CVB vaccine for clinical use has recently commenced. Phase I clinical trials began in December 2020 testing the safety and efficacy of this vaccine in humans (NCT04690426). Data indicates that the vaccine met primary safety endpoints and secondary efficacy endpoints (Provention Bio, 2021). If approved, this vaccine will initially be tested for the prevention of acute CVB infections and the delay or prevention of type 1 diabetes and celiac disease. Given that CVBs cause respiratory illness, the vaccine could also be relevant in CF populations.

### Limitations of the study

Finally, we acknowledge some limitations of our studies. First, we only examined the prevalence of CVB infections in a Swedish CF cohort and our observations must be confirmed in other CF populations. However, given the global prevalence of CVBs, one can assume that CVB infections are common in other CF populations. Secondly, we did not have access to childhood poliovirus vaccination records for the study participants, but during the last few decades in Sweden, poliovirus vaccine coverage has been around 97–98% therefore it can be assumed that most individuals were vaccinated in childhood. Whether individuals received a booster dose is unknown. It remains reassuring that most individuals had virus nAB titers that correlate with protection against infection. Thirdly, our proof-of-concept studies were limited to examining one CF-causing mutation, F508del, and our study involved an insufficient number of individuals to address whether different CFTR mutations affect poliovirus nAB responses. Fourthly, our pre-clinical testing of the CVB vaccines consisted of vaccinations with monovalent vaccines rather than a hexavalent CVB1-6 vaccine. In previous studies, we have found that mice respond equally well to monovalent CVB vaccines as to the polyvalent CVB vaccine (Larsson et al., 2015; Stone et al., 2018, 2020). It is therefore likely that a multivalent vaccine would also be efficacious in the F508del model.

### Conclusions

In summary, this study shows that CVB infections are common in the CF population and we provide pre-clinical evidence that CVB infections are preventable through vaccination. As respiratory virus infections are strongly linked to exacerbated pulmonary morbidity in CF and vaccines preventing such infections are advocated in CF, we propose that the role of CVB infections in pulmonary exacerbations should be further studied. If additional evidence supports the role for CVB infections in facilitating bacterial colonization and/or accelerating pulmonary morbidity in CF, the newly developed CVB vaccine could be a candidate for future prophylactic intervention in this disease.

## STAR★Methods

### Key resources table

**Table:** 

REAGENT or RESOURCE	SOURCE	IDENTIFIER
**Antibodies**		

FITC anti-mouse CD4	Biolegend	Clone RM4-5; Cat#100510; RRID:AB_312713
PE anti-mouse TCR beta chain	Biolegend	Clone H57-597; Cat#109207; RRID:AB_313430
APC anti-mouse/human CD45R/B220	Biolegend	Clone RA3-6B2; Cat# 103212; RRID:AB_312997
APC anti-mouse CD8a	Biolegend	Clone 53-6.7; Cat#100712; RRID:AB_312751

**Bacterial and virus strains**		

CVB1 (neutralizing antibody assay, Finland)	VacTech Oy, Finland	V200WE3C
CVB2 (neutralizing antibody assay, Finland)	VacTech Oy, Finland	V166XE4c
CVB3 (neutralizing antibody assay, Finland)	VacTech Oy, Finland	V13D4c
CVB4 (neutralizing antibody assay, Finland)	VacTech Oy, Finland	VD33SD4c
CVB5 (neutralizing antibody assay, Finland)	VacTech Oy, Finland	V10D3c
CVB6 (neutralizing antibody assay, Finland)	VacTech Oy, Finland	V147UD3c
Polio 1, Sabin (neutralizing antibody assay, Finland)	Terveyden ja hyvinvoinnin laitos – THL, Finland	MOPV1
Polio 3, Sabin (neutralizing antibody assay, Finland)	Terveyden ja hyvinvoinnin laitos – THL, Finland	MOPV3
CVB3-Nancy (neutralizing antibody assay, Sweden and vaccine, Finland)	Dr Gun Frisk, Uppsala University, Uppsala, Sweden	
CVB1-10796 wild-type strain (Argentina; vaccine and mouse infection studies)	CDC, USA	
CVB3 wild-type strain (Finland; vaccine)	Hus lab, Helsinki, Finland	

**Biological samples**		

Human serum (CF patients)	Stockholm CF Center, Karolinska University Hospital, Huddinge	n/a
Human serum (healthy controls)	Unrelated vaccine studies performed at Center for Infectious Medicine, KI	n/a

**Chemicals, peptides, and recombinant proteins**		

Coxsackievirus B1 antiserum	ATCC	V-028-501-560
Coxsackievirus B2 antiserum	ATCC	V-029-501-560
Coxsackievirus B3 antiserum	ATCC	V-030-501-560
Coxsackievirus B4 antiserum	ATCC	V-031-501-560
Coxsackievirus B5 antiserum	ATCC	V-032-501-560
Coxsackievirus B6 antiserum	ATCC	V-033-501-563

**Experimental models: Cell lines**		

Green monkey kidney (GMK) cells	National Institute for Health and Welfare, Finland	RRID:CVCL_L878
HeLa cells	Dr. R Glas, Center for Infectious Medicine.	RRID:CVCL_0030
Vero cells	ATCC	RRID:CVCL_0059

**Experimental models: Organisms/strains**		

B6.129S2-Tcra^*tm1Mom*^/J	Jackson Laboratories	RRID:IMSR_JAX:002115
C57Bl/6J strain 000664	Jackson Laboratories	RRID:IMSR_JAX:000664
Cftr^*tm1EUR*^	Dr Bob Scholte; Erasmus University, Rotterdam, The Netherlands	MGI:1857899

**Oligonucleotides**		

Primer TCRα KO mice genotyping mutant reverse: CTA CCC GCT TCC ATT GCT C	Invitrogen Custom Primers	Jackson Laboratories
Primer TCRα KO mice genotyping common: TGA CTC CCA AAT CAA TGT GC	Invitrogen Custom Primers	Jackson Laboratories
Primer TCRα KO mice genotyping wild-type reverse: GGT GAG ATG ACC CAA AGC AG	Invitrogen Custom Primers	Jackson Laboratories
Primer F508del mice genotyping CF-P581: CAC AAC ACT GAC ACA AGT AGC	Invitrogen Custom Primers	van Doornick et al., 1995
Primer F508del mice genotyping CF-P580: GGA CGC AAA GAA AGG GAT AAG	Invitrogen Custom Primers	van Doornick et al., 1995

**Software and algorithms**		

GraphPad 8.3.1	Graphpad	www.graphpad.com
FlowJo	FlowJo	www.flowjo.com
BioRender	BioRender	www.biorender.com

### Resource availability

#### Lead contact

Further information and requests for resources and reagents should be directed to the lead contact and will be fulfilled upon reasonable request, Malin Flodström-Tullberg (malin.flodstrom-tullberg@ki.se).

#### Materials availability

This study did not generate new unique reagents.

### Experimental model and subject details

#### Animals

Animals were randomly assigned to treatment groups. Researchers were not blinded to treatment groups during the experiment. Experiments with animals were performed in a BSL2 facility and mice were allowed to acclimatize to the room for a few days prior to the studies commencing. Extensive health monitoring of mice was performed throughout the studies including weekly weight measurements. Mice that lost >10% of their highest body weight or scored 0.4 or more points after health assessment were removed for ethical reasons. All animals were housed in a specific pathogen-free facility at Karolinska University Hospital Huddinge in isolated cages (maximum 5 animals per cage, with no single-housing of individual mice). The animals were housed, and experiments performed according to the local and national regulations. To try and minimise confounding factors, treatments were carried out at the same time of day where possible, the order in which cages were treated remained the same throughout the study and animal cages were kept in the same position throughout the study. Sample sizes in the different experiments were based on previous studies that used the same type of vaccines in other murine strains.

Two male mice homozygous for the Tcra^tm1Mom^ targeted mutation on a C57Bl/6J background (TCRα-knock out; B6.129S2-Tcratm1Mom/j) and four female C57Bl/6J wild-type mice (stock No: 000664; Charles River, Germany) were purchased from Jackson Laboratories. The female mice were hormone-treated and bred with the male animals; subsequently embryos were collected and implanted into foster mothers to allow for embryo rederivation ensuring a specific pathogen-free state. The resulting pups were genotyped using the following primers (5′ to 3′): mutant reverse – CTA CCC GCT TCC ATT GCT C; common – TGA CTC CCA AAT CAA TGT GC; wild type reverse GGT GAG ATG ACC CAA AGC AG according to the Jackson Laboratories protocol. Mice were bred as homozygotes (e.g. wt males with wt females or knock-out males with knock-out females) and genotyped as before. Female ko mice and wt controls between 9 and 20 weeks of age were included in the experiment.

The Cftr^tm1EUR^ mouse model (Rotterdam, the Netherlands), carrying the homozygous F508del mutation in the *Cftr* gene on a C57Bl/6J background was used (van Doorninck et al., 1995). Female and male transgenic (F508del) mice and their wt littermate controls aged between 4 and 22 weeks were included in the experiments. Mice were embryo-derived before being transferred into the animal facility, bred as heterozygotes and genotyped as previously described (van Doorninck et al., 1995).

The animal studies were approved by the Stockholm Southern Animal Ethics Board. The studies were performed in accordance with national and institutional guidelines.

#### Human subjects

Serum from a Swedish cohort of individuals with CF and from healthy controls was collected for the analysis of neutralizing antibodies against the six known CVB serotypes and poliovirus 1 and 3. All individuals with CF (n = 65) were monitored at Stockholm CF Center, serum dating between 1992 and 2010 was obtained from the clinic’s biobank. Serum from healthy individuals from the Stockholm region (n = 33) was obtained from two unrelated vaccination studies conducted between 2008 and 2016. Table 1 contains the characteristics (age, gender and CF genotype) of the CF and control patient cohorts. All healthy donors and individuals with CF gave informed consent prior to participation and experiments were conducted according to the Declaration of Helsinki. The studies were approved by the regional ethical review board in Stockholm, Sweden and were performed in accordance with national and institutional guidelines.

#### Microbe strains

For vaccine production, CVB3 (wild-type strain from Finland; (Laitinen et al., 2014)), CVB3 Nancy (kindly provided by Dr. G. Frisk, Uppsala University) and CVB1-10796 (wild -type strain from Argentina (Hamalainen et al., 2014)) were propagated in Vero cells (National Institute for Health and Welfare, Finland) and recovered from supernatant as described in Hankaniemi et al. (2019). In infection studies, animals were challenged with CVB1-10796 or 10^5^ PFU CVB3-Nancy (both propagated in HeLa cells).

#### Cell lines

Vero cells (RRID:CVCL_0059, female, ECACC no. 84113001, mycoplasma negative) were propagated in DMEM containing 10% FBS, 2mM L-Glutamine and 1% penicillin-streptomycin. GMK cells (RRID:CVCL_L878; gender unknown; mycoplasma negative) were cultured in Eagle’s minimum essential medium supplemented with 10% FBS. HeLa cells (RRID:CVCL_0030; female; mycoplasma negative) were grown in RMPI containing 10% FBS and 2mM L-Glutamine. None of the cell lines were authenticated.

### Method details

#### Flow cytometry analysis

Flow cytometry analysis was performed to assess the presence of T- and B-cells in splenocytes from the wt and TCRα knock-out mice. Briefly, spleens were harvested, homogenised and then the homogenate was collected and pelleted. Red blood cells in the pellet were lysed and the cells were washed, then resuspended in FACS buffer (PBS +2% fetal bovine serum + 2mM EDTA) and counted. Cells (10^6^ cell/ml) were stained with anti-CD4, anti-TCRβ or anti-B220 antibodies (all from Biolegend; 1:400 dilution) and then analysed by flow cytometry using an Accuri Flow Cytometer (BD). 10,000 events were assessed per sample and the data was analysed using FlowJo software.

#### Neutralizing antibody measurements

Measurements of serum neutralizing antibodies towards the six CVB serotypes and polio 1 and 3 in human serum samples and CVB1 in mouse samples were performed at Tampere University, Finland, using a standard plaque reduction assay (Laitinen et al., 2014). Briefly, serum samples were diluted by serial fourfold dilutions (1:4 and 1:16 for CVB1-6 wild-type strains and 1:4–1:16384 for polio 1 and 3 Sabin strains) and then incubated with infectious virus (CVB1-6 or polio 1 or 3, approximately 100 plaque forming units (PFU)) for one hour in at 37°C followed by an overnight incubation at room temperature. Samples were then further diluted with HBSS-HEPES (Gibco), added to 95% confluent GMK cell monolayers and incubated for 30 minutes at 37°C. The serum dilutions were removed, the cells were covered with semisolid media (MEM containing 0.66% Carboxymethyl Cellulose, Sigma) and incubated for two days in at 37°C, 5% carbon dioxide. Cells were then fixed with 4% formaldehyde solution containing 0.25% Crystal Violet (Electron Microscopy Sciences) for visualization and quantification. A sample with reduction of plaque number ≥80% compared to pure virus suspension was considered positive for neutralizing antibodies. The detection limit of the assay was a fourfold dilution 1:4 and the protective titre for human serum was defined as ≥ 1:16 for CVB1-6 and ≥ 1:8 for polio 1 and 3 (O'Ryan et al., 2015). For CVB1-6, positive control wells in which virus infectivity was neutralized using specific horse antiserum to CVB1-6 (ATCC, VR-1032-33-34-35-36-37AS/HO) were included in each test run.

Neutralizing antibody titres against CVB3 in mouse serum samples were analyzed by standard plaque neutralization assay at the Karolinska Institutet, Sweden. The serum was serially diluted by 10-fold dilutions (10^−1^ – 10^−3^) in RPMI 1640 media (ThermoFisher). Serum was then mixed with CVB3 (Nancy strain; 30 plaque forming units (PFU)) diluted in RPMI 1640 media and incubated for one hour at 37°C in a standard cell incubator followed by overnight incubation at room temperature. The following day a standard plaque assay was performed. Briefly, the serum-virus dilutions were applied to confluent HeLa cell monolayers and incubated for one hour at 37°C with tilting every 15 minutes. The serum-virus dilutions were then removed, and the HeLa cell monolayers were covered with 3 mL of 2% of agarose solution (low melting point, Sigma) supplemented with 50% 2xMEM (ThermoFisher) and 10% fetal calf serum. The plates were incubated for three days at 37°C in a standard incubator and thereafter fixed with Carnoy’s Reagent (methanol:acetic acid, 3:1) for one hour. The Carnoy’s solution and agarose plugs were then removed; the plates were stained with 0.2% Crystal Violet and washed with water, after which the plaques were counted. A sample with a reduction in plaque number by ≥ 80% compared to the virus control (30 PFU) was considered positive for neutralizing antibodies against CVB3.

#### Vaccine production

CVB3 (wild-type strain from Finland; (Laitinen et al., 2014)), CVB3 Nancy (kindly provided by Dr. G. Frisk, Uppsala University) and CVB1-10796 (wild -type strain from Argentina (Hamalainen et al., 2014)) were propagated in Vero cells (National Institute for Health and Welfare, Finland) and recovered from supernatant as described in Hankaniemi et al. (2019). Briefly, infected cell supernatant was used to recover the viruses by 30% sucrose cushion pelleting (175000g, 6 to 16 hours at 4°C). Collected pellets were resuspended in PBS-0.1% Tween and further purification was performed with gelatin affinity chromatography resin (GE Healthcare). Virus was recovered from the flow-through. In the last step, the virus samples were pelleted through discontinuous 30/50% sucrose cushion ultracentrifugation (285000g, 14 hours at 4°C) and then the pellet was dissolved in PBS-0.1% Tween 80. The three CVB strains were inactivated in 0.01% (vol/vol) formalin for 5 days at 37°C. The vaccines were formulated in M199 medium (Gibco) containing 0.1% Tween80 by mixing 1.8 μg of protein per vaccine dose. Buffer control vaccinations were performed with the M199 medium containing 0.1% Tween80 (also used to dilute the vaccines). The vaccines were characterised to confirm their quality by SDS-Page (purity), BCA assay (protein concentration), Western blot (to detect viral proteins), dynamic light scattering (to assess particle size and distribution) and TCID_50_ assay (to confirm inactivation of the virus) as described in (Stone et al., 2020) (data not shown).

#### Vaccinations

Vaccination strategies were based upon those used in previous studies (Larsson et al., 2015; Stone et al., 2020). In all vaccination studies, mice were monitored by weight and observations of the overall health score (general state, movement, piloerection, skin fur and colour, respiration, and appetite). All vaccines contained 1.8 μg of protein per dose and were given by interscapular (subcutaneous) injection (total volume 150 μL).

Female TCRα-knock out (ko) and wild-type (wt) mice (9–20 weeks old) were vaccinated twice on days 0 and 14 with a monovalent CVB3 vaccine (wild-type strain). Serum was collected on days −2, 4, 5, 14, 21, 28 and 35 after the initial vaccination for the measurement of neutralizing antibodies. Mice were euthanised on day 35 after the final serum sampling or earlier if the overall health score indicated that an animal was unwell.

Two vaccination schedules were employed for the F508del mice. To examine the early antibody responses to vaccination, female wt and F508del (4–22 weeks old) mice were vaccinated with two doses of monovalent CVB3 (wild-type strain) vaccine on days 0 and 14 and serum was collected on days −2, 4, 5, 14 and 28 after the initial vaccination for the measurement of neutralizing antibodies.

In studies examining the protective ability of CVB vaccines against virus infection in F508del mice, a three-dose vaccination schedule was followed. Briefly, female and male F508del mice were vaccinated on days 0, 21 and 35 with either CVB1 vaccine (wild-type strain; n = 6) or CVB3 (Nancy strain; n = 10) or left untreated (n = 1) or mock vaccinated with vaccine buffer (n = 6 for CVB1 studies; n = 3 for CVB3 studies). Serum was collected on days −4, 4, 21, 35 and 63.

#### Virus infection

Mice in the infection studies were challenged with 10^6^ PFU CVB1-10796 or 10^5^ PFU CVB3-Nancy or by intraperitoneal injection on day 63 after the initial vaccination. Unvaccinated (n = 1; CVB3) and mock vaccinated F508del mice (n = 3 for CVB3 studies; n = 6 for CVB1 studies) were infected with the same respective dose of CVB1 or CVB3. Blood was collected on days 3 and 4 post infection and mixed 1:1 with 12 mM EDTA. Mice were terminally anesthetized on day 67 and organs (pancreas, liver, lung, spleen, heart) were removed, divided and frozen for later measurement of viral load by standard plaque assay or fixed with 4% paraformaldehyde (PFA) for histological evaluation.

#### Measurement of viral titres

CVB1 and CVB3 titers in blood and organs (pancreas, spleen, liver, lung and heart) were analysed by standard plaque assay as described above. Blood was serially diluted 20^−1^ to 20^−6^ in RPMI media. Organs were homogenised using a TissueLyser II (Qiagen, Sollentuna, Sweden) in 1mL of RPMI media and serially diluted 10^−1^ to 10^−8^ in RPMI media.

#### Histological evaluation

Organs were fixed in 4% PFA solution at room temperature overnight, dehydrated, embedded in paraffin blocks, and cut into 5 μm thick sections. Sections were stained with Mayer’s haematoxylin and Eosin Y and mounted with Pertex (Histolab products AB, Askim, Sweden). All organs were scored by researchers who were blinded for both experimental design and animal identity. Pancreas sections were scored according to immune infiltrate and exocrine destruction. Sections of spleen were assessed by the integrity of the red and white pulp. Liver samples were assessed according to organ integrity and the presence or absence of pathological signs including ballooning degeneration and steatosis. Lungs were scored by ordinal degree for perivascular and peribronchial inflammation, fibrosis, parenchymal and alveolar damage, red blood cell infiltrate, and general overall health status (0–4; rare, multifocal, coalescing, diffuse; (Gibson-Corley et al., 2013)). In the case of the lungs, three sections from three different levels (minimum 50 μm between even level) per animal were scored.

### Quantification and statistical analysis

Statistical analyses were performed using GraphPad 8.3.1 (San Diego, USA). Differences in categorical variables such as gender (Table 1) were determined using a chi square test. Differences in continuous variables between two groups, e.g. CF and healthy individuals (Table 1), and in viral titres in the blood and organs of vaccinated mice were (Figure 4) were tested using a Mann-Whitney U test, while comparisons between more than two groups, e.g. plaque neutralisation assay data (Figure 1), were performed using ANOVA with Kruskal-Wallis test. Comparisons between CVB3 neutralizing antibody titres in wt and F508del/TCRα-ko mice (Figures 2, Figure 3) and differences in body weight changes (Figure 4) were performed by two-way ANOVA with Sidak’s multiple comparison test. Neutralizing antibody responses were visualised with an Akima spline (Figures 2 and 3). Pancreas and lung health scores (Figure 5) and poliovirus neutralizing antibody titres (Figure 6) were compared by an unpaired t-test. Data is represented as the mean ± standard deviation, with individual values also shown. *p*-values < 0.05 were considered significant. N numbers are given in the figure legends (n denotes the number of individuals/animals analysed).

## Data Availability

• All data reported in this paper will be shared by the lead contact upon request. • This paper does not report original code. • Any additional information required to reanalyse the data reported in this paper is available from the lead contact upon request.
