# Supplementary material for: Coxsackievirus B infections are common in Cystic Fibrosis and experimental evidence supports protection by vaccination
Source: iScience. 2022 Sep 5;25(10):105070. doi: 10.1016/j.isci.2022.105070 (PMC9490033; doi:10.1016/j.isci.2022.105070)
Supplement: Document S1. Figures S1–S6 [file mmc1.pdf]

iScience, Volume *25*

Supplemental information

Coxsackievirus B infections are common in Cystic Fibrosis and experimental evidence supports protection by vaccination

Virginia M. Stone, Renata Utorova, Marta Butrym, Amir-Babak Sioofy-Khojine, Minna M. Hankaniemi, Emma E. Ringqvist, Marfa Blanter, Anirudra Parajuli, Terezia Pincikova, Björn Fischler, Ferenc Karpati, Vesa P. Hytönen, Heikki Hyöty, Lena Hjelte, and Malin Flodström-Tullberg

Supplementary Information

Figure S1: Seropositivity to the six CVB serotypes in individuals with CF and controls, related to Figure 1. 2

Figure S2. TCRα knock-out mice lack TCRαβ cells, related to Figure 2. 3

Figure S3. CVB3 vaccination does not alter weight gain in TCRα ko and wt mice and in F508del animals, related to Figure 2. 4

Figure S4. CVB1 and CVB3 vaccination does not alter weight gain in F508del animals, related to Figure 3. 5

Figure S5. F508del mice vaccinated with CVB1 and CVB3 vaccines are protected from CVB mediated pathology in the pancreas and lung, related to Figure 5. 6

Figure S6. CVB vaccines prevent CVB-induced liver pathology in F508del mice, related to Figure 5. 7

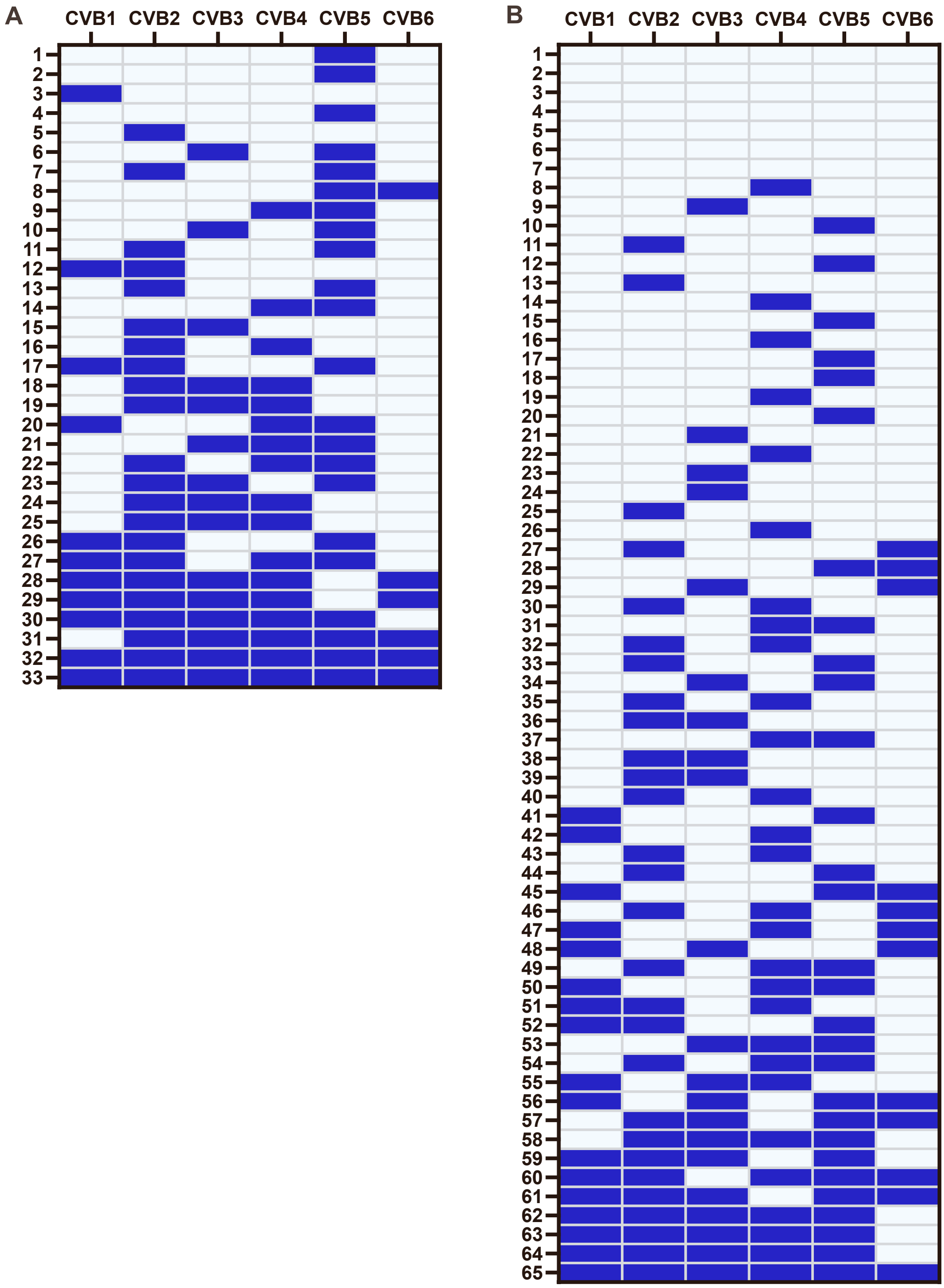

Figure S1: Seropositivity to the six CVB serotypes in individuals with CF and controls, related to Figure 1. Serum collected from healthy controls (**a**; n=33) and individuals with CF (**b**; n=65) was assessed for neutralising antibodies against the six CVB serotypes by standard plaque neutralisation assay. Dark rectangles illustrate individuals positive for the respective CVB serotype and the light rectangles represent negative samples.

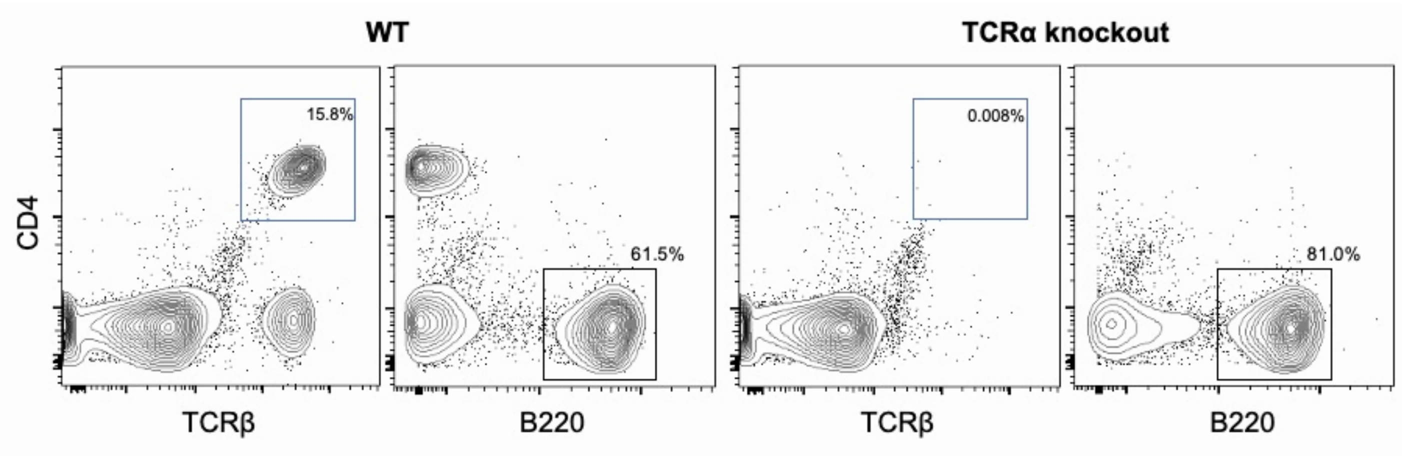

Figure S2. TCRα knock-out mice lack TCRαβ cells, related to Figure 2. Splenocytes were isolated from male wild-type (wt) and TCRα knockout (ko) mice, stained with the T-cell specific markers CD4 and TCRα and the B cell marker B220 and then analysed by flow cytometry. In total splenocytes were isolated, stained and analysed from one wt and two heterozygote mice (controls) and two ko mice. Data shown are representative plots from one wt and one ko animal. The blue boxes represent $CD4^{+}TCR\beta^{+}$ T cells and the black boxes show the $B220^{+}$ B-cell population. Cell percentages are given next to each box.

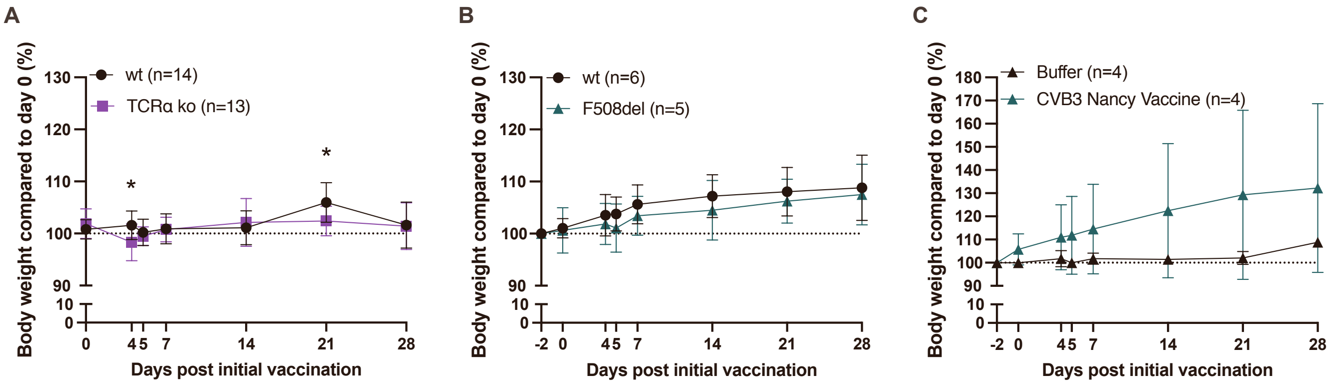

Figure S3. CVB3 vaccination does not alter weight gain in TCRα ko and wt mice and in F508del animals, related to Figure 2. (a) Female wt (n=14) and TCRα ko (n=13) mice were vaccinated on two occasions with monovalent CVB3-field isolate vaccine (day 0 and 14; 1.8 μg per dose; interscapular injection) and weight was monitored until day 28 after the first vaccination. Data is shown as mean percentage bodyweight change compared to day -2 (the dotted line) ± SD. (**b** and **c**) Female and male F508del and wt mice were vaccinated with either CVB3-field isolate vaccine (b; wt =6; F508del = 5), CVB3-Nancy vaccine (C; F508del mice only n=4) or vaccine buffer (c; F508del only, n=4) on days 0 and 14 (1.8 μg per vaccine dose; interscapular injection) and weight was monitored until day 28 after the first vaccination. Data is shown as mean percentage bodyweight change compared to day 0 (the dotted line) ± SD. * $p < 0.01$ comparing wt and TCRα ko mice at each time point by two-way ANOVA with Sidak's multiple comparison test.

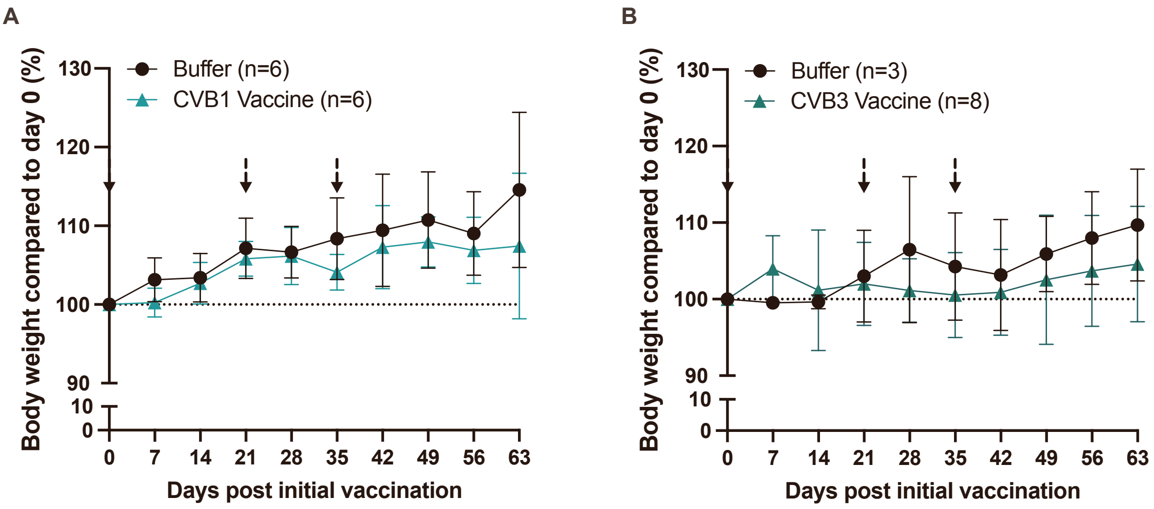

Figure S4. CVB1 and CVB3 vaccination does not alter weight gain in F508del animals, related to Figure 3. (a and **b)** Female and male F508del mice were vaccinated on three occasions with either CVB1 vaccine (a; n=6), CVB3-Nancy vaccine (b; n=8) or were mock vaccinated with vaccine buffer (a; n=6; b; n=3) on days 0, 21 and 35 by interscapular injection. Each vaccine dose contained 1.8 μg of protein. Body weight was monitored up until the point of infection (day 63 after the initial vaccination). Data is shown as mean percentage bodyweight change compared to day 0 (the dotted line) ± SD. The arrows indicate the days when the animals were vaccinated. No significant differences were detected between the buffer and vaccine groups at each time point (two-way ANOVA with Sidak’s multiple comparison test).

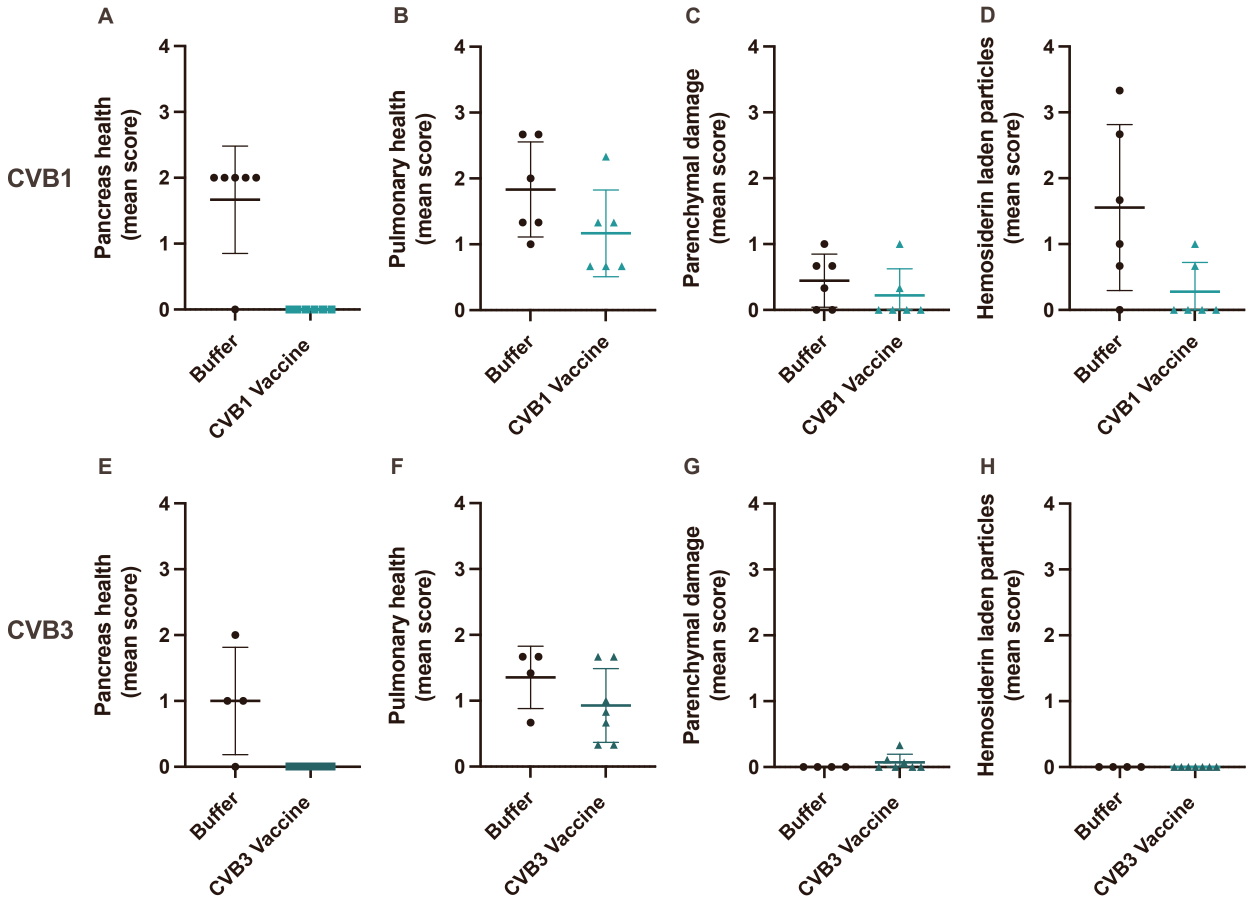

Figure S5. F508del mice vaccinated with CVB1 and CVB3 vaccines are protected from CVB mediated pathology in the pancreas and lung, related to Figure 5. Female and male F508del mice were left untreated (n=1 for CVB3 group), buffer treated (n=6 for CVB1 group and n=3 for CVB3 group) or vaccinated with CVB1 (n=6) or CVB3 (n=8) vaccines on days 0, 21 and 35 (1.8 μg per dose; interscapular injection) and infected with CVB1 or CVB3 virus on day 63 as shown in Fig. 3a. Organs were collected on day 4 post infection for histological analysis of organ integrity by haematoxylin and eosin staining of formalin fixed paraffin embedded sections. (**a** and **e**) Pancreas scores of buffer + CVB or vaccine + CVB groups infected with (a) CVB1 or (e) CVB3. Each pancreas was scored according to exocrine damage and the presence of infiltrating immune cells. (**b** and **f**) Pulmonary health mean score, (**c** and **g**) parenchymal damage mean score and (**d** and **h**) hemosiderin laden particles mean score in the lungs of buffer treated + CVB infected or vaccinated + CVB infected mice infected with CVB1 (b – d) or CVB3 (f– h). For (a – c and e – g) a score of 0 indicates healthy tissue and a score of 4 indicates highly damaged tissue. Each mouse is represented by an individual symbol and shown are the mean values ± SD.

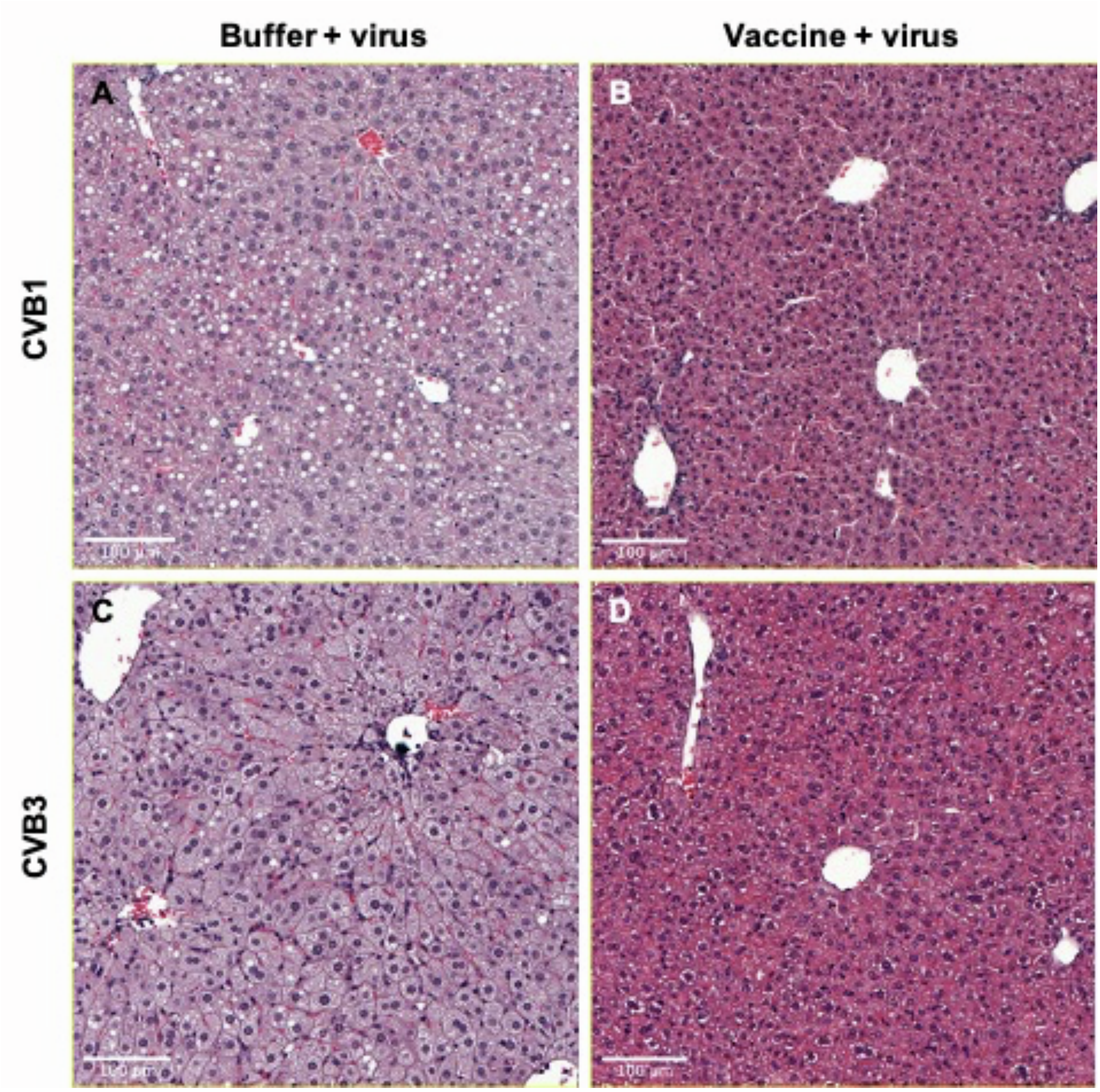

Figure S6. CVB vaccines prevent CVB-induced liver pathology in F508del mice, related to Figure 5. Female and male F508del mice were left untreated, buffer treated (**a** and **c**) or vaccinated with CVB1 (**b**) or CVB3 (**d**) vaccines on days 0, 21 and 35 (1.8 μg per dose; interscapular injection) and then infected with CVB1 (**a** and **b**) or CVB3 (**c** and **d**) on day 63 as shown in Figure 3a. Livers were collected on day 4 post infection, formalin fixed and paraffin embedded then sectioned for histological analysis of organ integrity by haematoxylin and eosin staining. Representative images from the buffer and vaccine groups are shown.
